# Characterising complex metabolic responses in an engineered, cross-feeding microbial co-culture using quantitative proteomics

**DOI:** 10.1016/j.nbt.2026.02.001

**Published:** 2026-07-25

**Authors:** Mengxun Shi, Josie McQuillan, Caroline Evans, Yanmeng Liu, Xiaoxia Nina Lin, Brett Barney, Jagroop Pandhal

**Affiliations:** a School of Chemical, Materials and Biological Engineering, The University of Sheffield, Sheffield S1 3JD, UK; b Department of Chemical Engineering, University of Michigan, Ann Arbor, MI 48109, USA; c Department of Bioproducts and Biosystems Engineering, University of Minnesota, St. Paul, MN, USA

**Keywords:** Co-culture, Proteomics, Synechococcus, Azotobacter, Cross-feeding

## Abstract

Microbial communities play a key role in biogeochemical transformations in a wide range of ecosystems, but they also hold significant potential to enhance the bioproduction of desired chemicals. Although designing synthetic microbial consortia has generated a lot of interest, a more in-depth understanding of the interactions between strains is required, particularly when strains are engineered to cross-feed, but are not isolated from related environments. Challenges include enhancing stability, productivity and controllability. Here, we used a synthetic microbial co-culture consisting of engineered strains of the photosynthetic cyanobacterium *Synechococcus elongatus* PCC 7942 cscB/SPS and nitrogen-fixing bacterium *Azotobacter vinelandii* AV3. Each relies on the other for conversion of atmospheric carbon (CO_2_) and nitrogen (N_2_) into organic forms, *i.e.* sucrose and ammonia, respectively, resources which can be shared. As both strains have such contrasting growth dynamics in co-culture compared to monoculture, we applied a label-free quantitative proteomics approach to characterise metabolism in both strains. The proteomes of both shifted when in co-culture to reflect adaptive restructuring of carbon and nitrogen metabolism, although *A. vinelandii* appeared to transition to a more stressed state, inducing proteins linked to polymer biosynthesis. An analysis of the co-culture over 16 days led to phenotypic changes, including cell structure alterations in *A. vinelandii* AV3 over time, with the proteome suggesting cell envelope remodelling and potentially encystment. These findings suggest that physiological control of parameters, such as oxygen and nutrient availability, may enable cultivation of more stable co-cultures.

## Highlights

• Quantitative proteome investigation of synthetic microbial co-culture.
• Cross-feeding of organic carbon and nitrogen.
• Evidence of specific stress responses in co-culture.
• Key insights into strategies for process optimization.

## 1 Introduction

Advances in microbial fermentation have led to a wider variety of bio-based products [1], and with the advent of emerging synthetic biology tools, efforts to optimise production of more tightly-regulated or novel compounds are on the increase. However, as engineered metabolic pathways introduced into microbial chassis become more complex, slower growth rates and reduced titres frequently impair the overall effectiveness and viability of industrial processes [2], [3]. The development of synthetic microbial communities is an innovative new strategy to address these issues [4], [5].

Microbial communities, ubiquitous in nature, are groupings of two or more microorganisms that live in the same environment. They can perform more complicated metabolic activities than monocultures, requiring pools of specific precursors or specialised compartments, and withstand more changeable environments due to their distinct features [6]. Members in microbial communities can interact with each other via the exchange of specialised chemical signals or metabolite transactions. Moreover, labour division among different individuals make co-cultures valuable in bioproduction [7]. For example, Eiteman et al., developed a system comprising two *Escherichia coli* strains; one that preferentially consumes glucose whilst the other only consumes xylose, to metabolise the substrates more efficiently in the co-culture [8]. In another study, Zhou et al. created two distinct synthetic pathways to produce an acetylated diol paclitaxel precursor, a drug utilised to treat a range of cancers, but rarely seen in nature. Each pathway was expressed in separate host cells suited to their bioproduction, *Saccharomyces cerevisiae* and *E. coli* [9]. In other studies, when the feedstock frequently varies in composition, such as in wastewater treatment, microbial communities are usually more effective compared to monocultures [10]. Natural microbial communities have been used in industrial processes for years [11], [12]; however, getting them to perform more complex or bespoke tasks in biomanufacturing or resource recovery is more of a challenge, and therefore the rapid development of synthetic biology and omics tools have enabled advanced characterisation of strains, and ultimately the design of synthetic consortia.

Integrated omics techniques including genomics and transcriptomics are universal approaches for species analysis, which have been used to study environmental communities [13], [14] and human microbiomes [15], [16]. However, their interpretation is limited by the fact that genomic information doesn’t equate to function, and mRNA abundance provides only a prediction of protein abundance and activity [17]. Hence, there are gaps in knowledge of the mechanisms of translational control in dynamic and interacting microbial communities. For these reasons, more functionally relevant workflows in quantitative proteomics and metabolomics are highly valued. With the development of high accuracy mass spectrometry technology and advances in bioinformatics tools, mass spectrometry-based proteomics has become widely used for the identification, characterisation, and quantification of proteins.

Quantitative proteomics can provide functional insight into co-cultures, with information on both species. This approach has been applied with samples sourced from the natural environment [18], [19], gut bacteria [20], [21], and health-related research [22]. With new approaches for dealing with challenges with processing mixed communities, such as missing intensity values and normalising dynamic changes in cell ratios [23], [24], the potential to investigate interactions with the view to improve stability and productivity characteristics has grown. We applied a quantitative proteomics workflow to investigate the interactions within an engineered cross-feeding microbial co-culture: i) *Synechococcus elongatus* PCC 7942 cscB/SPS, a photosynthetic freshwater cyanobacterium reported in Abramson *et al.* (2016), which is a derivative of *S. elongatus* PCC 7942, generated by integrating sucrose permease (cscB) from *Escherichia coli* and overexpressing sucrose phosphate synthase (SPS) from *Synechocystis* sp. PCC 6803 [25], and ii) *A. vinelandii* AV3, a nitrogen-fixing bacterium, which is a derivate of *A. vinelandii* DJ, produced by knockout of the nifL gene, a repressor of nitrogenase [26]. First established by Smith and Francis in 2016, in this system the sucrose produced by *S. elongatus* acts as the organic carbon source for *A. vinelandii* AV3, and ammonium produced by *A. vinelandii* AV3 provides organic nitrogen for *S. elongatus* [27]. Therefore, this synthetic microbial co-culture can utilise CO_2_ and N_2_ in the air as carbon and nitrogen sources. In their study, variations in initial cell ratios and density were investigated, and showed promising results, although growth rates tend to stabilize to stationary phase within 2–3 days [27].

In a previous proteomics study on a eukaryotic alga and bacterium co-culture, we were able to propose that despite stability in the co-culture, metabolic trade-offs are required to minimize stress and promote mutualism in the alga [28]. However, the response of the bacteria was not investigated. In a more recent study, we applied our quantitative proteomics pipeline to a co-culture of the same engineered *S. elongatus* strain co-cultivated with soil bacterium *Pseudomonas putida* cscRABY [29]. This *Pseudomonas* strain had been modified to transport and metabolise sucrose, and in fact has a growth promoting effect on *S. elongatus.* The multi-omics approach suggested specific stress responses, as well as dynamic changes in transport mechanisms, pointing to the importance of media chemistry when co-culturing microbes. In this study, we aimed to better understand the growth dynamics when co-cultivating *A. vinelandii* and *S. elongatus* in batch culture, in a specific media. By applying a novel quantitative proteomics workflow, we aimed to understand how metabolism is affected and potential interaction mechanisms. The protein changes were compared between the monocultures and co-culture at a specific time point when both strains were interacting at similar cell densities, and subsequently in a time series co-culture experiment, where morphological changes implied rewiring of metabolic processes, revealing complex interactions amongst expected macronutrient exchange processes.

## 2 Materials and methods

### 2.1 Co-culture setup

*S. elongatus* PCC 7942 cscB/SPS (herein referred to as *S. elongatus*) stocks were cultured in BG11 medium [30] with 50 μg/mL kanamycin and 25 μg/mL chloramphenicol. *A. vinelandii* AV3 (herein referred to as *A. vinelandii*) stocks were cultured in Burk’s N-free medium [31]. After growing to early mid-log phase for 7 days, *S. elongatus* was washed with phosphate buffered saline (PBS) and inoculated into SAC medium (pH 7.3) (composition shown in Supplementary files, Table S1, SAC7), with 0.5 mM IPTG to induce sucrose production. IPTG-induced *S. elongatus* was cultured axenically in SAC medium for two days to an OD_750_ of 0.746 before inoculation with *A. vinelandii*. An initial cell ratio of 20:80 of *A. vinelandii* to *S. elongatus* was applied to establish the co-culture. The *S. elongatus* and *A. vinelandii* co-cultures were cultured in 500 mL flasks with 250 mL SAC medium at 30 °C under constant light intensity at 120 µE·m^−2^·s^−1^ with shaking at 120 rpm. Control *S. elongatus* and *A. vinelandii* monocultures were cultivated under the same conditions as the co-cultures. Atmospheric CO_2_ and N_2_ were used as carbon and nitrogen sources; flasks were covered with permeable membranes to facilitate gas exchange. Each experiment was repeated in three biological replicates.

### 2.2 Cell counting

*S. elongatus* cells were counted using an A60-Micro PLUS flow cytometer (Apogee Flow Systems, Hemel Hempstead, UK) equipped with 405 nm, 488 nm and 561 nm diode lasers. Three photomultiplier tubes were installed to collect small-angle light scatter, medium-angle light scatter and large-angle light scatter signals. Calibration was performed using 110–1300 nm diameter silica beads (ApogeeMix, #1493). Cells were measured using 405-MALS (325 V) and 561 nm orange (500 V) lasers. Data were acquired at a flow rate of 1.5 μL/min with a sample volume of 130 μL under the sheath fluid pressure of 150 mbar and recorded using Histogram software (Apogee Flow Systems).

*A. vinelandii* cell counting was achieved by measuring colony-forming units (CFUs). Briefly, 100 μL of cell culture was added to 900 μL Burk’s N-free medium to make dilution 1 (10^−1^). The same dilution method was repeated to prepare dilution 2 (10^−2^), dilution 3 (10^−3^), and dilution 4 (10^−4^). Then, 10 μL from dilutions 2, 3 and 4 were plated onto Burk’s N-free agar plates supplemented with 25 mg/L bromothymol blue to facilitate colony counting. The plates were incubated at 30 °C for three days. Three biological replicates and two technical replicates were performed for each experiment. CFUs were converted to cell numbers using a CFU-to-cell number standard curve.

The two organisms were quantified using different methods due to intrinsic biological differences and measurement limitations. Due to autofluorescence and stable single-cell morphology, *S. elongatus* was enumerated by flow cytometry for accurate total cell counts. In contrast, *A. vinelandii* can exhibit morphological changes during co-culture (see Supplementary Files S5), particularly under stress, and therefore viable *A. vinelandii* cells were quantified using CFU-based plate counts. These were deemed suitable for comparing relative growth dynamics.

### 2.3 Measurement of sucrose and ammonium production

Sucrose concentration was determined using a Sucrose/ D-Glucose Assay Kit according to the manufacturer’s instructions (K-SUCGL, Megazyme). Ammonia concentration was determined using the modified Nessler method [32]. Samples were diluted with ammonia-free water (VWR Chemicals). To 1 mL diluted samples, 20 µL of mineral stabilizer solution (HACH), 20 µL of 0.135 % (w/v) polyvinyl acetate (Sigma-Aldrich), and 40 µL of Nessler reagent (Sigma-Aldrich) were added and mixed using a vortex. Samples were incubated for 10 min at room temperature, then absorbance was measured at 425 nm against a standard curve.

### 2.4 Protein extraction, digestion and peptide preparation

Samples (25 mL) were harvested from each biological replicate at 5000 rpm, 4 °C for 10 min. Crude protein extraction was performed as previously described [33].

Crude protein samples were purified using 2D Clean-Up Kit according to the manufacturer’s instructions (GE Health, Buckinghamshire, UK). Protein concentrations were measured using BradfordUltra reagent (Expedeon, Cambridgeshire, UK) following the manufacturer’s instructions.

Protein lysates were digested as described previously [34], [35] with modifications. Briefly, cleaned-up protein pellets were dissolved in 30 µL urea buffer (8 M urea/ 100 mM Tris-HCl pH 8.5/ 5 mM DTT) followed by water bath sonication to fully suspend. Protein concentrations were determined using a NanoDrop™ 2000 spectrophotometer (Thermo Scientific, Delaware, USA). Approximately 50 µg of protein samples were diluted to 10 µL with urea buffer and incubated at 37 °C for 30 min (reduction step), then 1.5 µL of 100 mM iodoacetamide was added and incubated in the dark at room temperature for 30 min (alkylation step). MS grade trypsin (Promega, Wisconsin, USA) was added in a 1:50 (w/w) protease:protein ratio to the protein solutions, which were then diluted with 50 mM Tris-HCl (pH 8.5)/ 10 mM CaCl_2_ to a final concentration of 1 M urea. The protein solutions were incubated overnight at 37°C. Trypsin digestion was terminated by adding formic acid to a final concentration of 1 %. Digested peptides were desalted using Bond Elut OMIX C18 tips (Agilent Technologies) following the manufacturer’s instructions and dried by vacuum centrifugation (Eppendorf, Hamburg, Germany).

### 2.5 Shotgun LC-MS/MS analysis

Liquid chromatography-tandem mass spectrometry (LC-MS/MS) proteomic analysis was performed as previously reported [36] with modifications. Dried peptide pellets were dissolved in 50 µL loading buffer (3 % (v/v) acetonitrile; 0.1 % (v/v) trifluoroacetic acid) by sonication, then centrifuged at 13,000 rpm, 2 min. LC-MS/MS was performed using a nano-flow liquid chromatography (U3000 RSLCnano, Thermo Fisher Scientific, United Kingdom) coupled to a hybrid quadrupole-orbitrap mass spectrometer (Q Exactive HF, Thermo Fisher Scientific, United Kingdom). Peptides were separated on an Easy-Spray C18 column (75 μm × 50 cm) using a 2-step gradient from 3 % solvent A (0.1 % formic acid in water) to 10 % solvent B (0.1 % formic acid in 80 % acetonitrile) over 5 min and then to 50 % solvent B at 300 nL min^−1^, 40 °C. The mass spectrometer was programmed for data-dependent acquisition with 10 product ion scans (resolution 30,000, automatic gain control 1e5, maximum injection time 60 ms, isolation window 1.2 Th, normalized collision energy 27, and intensity threshold 3.3e4) per full MS scan (resolution 120,000, automatic gain control 1e6, maximum injection time 60 ms) with a 20-s exclusion time.

### 2.6 Protein identification and quantification

For protein identification of the monoculture and co-culture samples, a reference database was created using all protein sequences of *S. elongatus* PCC 7942 (2874 sequences) and *A. vinelandii* DJ (5013 sequences) appended with CscB from *E. coli* and SPS from *Synechocystis* sp. PCC6803 from Uniprot (https://www.uniprot.org/, Feb 2020), resulting in a final database of 7889 protein sequences. Raw MS data files were processed using MaxQuant (2.0.3.0) and its built-in Andromeda search engine for peptide identification and protein inference [37]. Default settings were used with search parameters set to include the following modifications: Oxidation (M) and Acetyl (Protein N-term) (variable); Carbamidomethyl (C) (fixed). Peptide-spectrum matches and protein identifications were filtered using a target-decoy approach at a false discovery rate (FDR) of 1 %. Label free quantification (LFQ) and intensity-based absolute quantification (iBAQ) options were selected [38].

### 2.7 Normalisation

To minimize the impact of cell number changes on differential expression analysis, normalisation was carried out on co-culture and monocultures based on the LFQRatio method (Eq. 1) we reported previously [23]. Briefly, for each individual protein detected, its LFQ intensity was divided by the sum of all protein LFQ intensities for its respective strain.

(1) $$\mathrm{Normalised}\;\mathrm{LFQ}\;\mathrm{intensity}=\frac{\mathrm{LFQ}\;\mathrm{intensity}\;\mathrm{of}\;\mathrm{one}\;\mathrm{protein}}{\mathrm{total}\;\mathrm{LFQ}\;\mathrm{intensities}\;\mathrm{of}\;\mathrm{the}\;\mathrm{strain}}\;$$

### 2.8 Differential expression, functional categories and pathway assignment analysis

Differential protein expression analyses comparing i) mono- and co-cultures and ii) co-cultures over time were performed using Amica v3.0.1 (https://bioapps.maxperutzlabs.ac.at/app/amica) [39]; normalised LFQ intensity values for proteins matched and quantified by MaxLFQ were uploaded for analysis. Analysis settings selected: min. razor peptides, 2; min. MSMS count, 3; Min. valid values in each group, 2; No normalization; No imputation; LIMMA-based DEP analysis. As no imputation was selected, any proteins completely absent from either condition compared were filtered out by Amica. For differential expression analysis: adjusted p-value cutoff, 0.05; log_2_ fold-change (Log_2_FC) cutoff, 1.

Gene ontology (GO) analysis of the differentially expressed proteins (DEPs) was performed using ShinyGO v0.85.1 (https://bioinformatics.sdstate.edu/go/) [40], using MaxQuant-identified proteins as the background environment for each organism.

### 2.9 Generating an *A. vinelandii* PHB/alginate mutant

To test the effects of β-polyhydroxybutyrate (PHB) and alginate production on ammonium accumulation, the strain AZBB764 (*Avin_11360*-*kan-pPCRNH3–44-imitation-nifA*-*Avin_11370*, Δ*nifLA*, Δ*amtB*, Δ*algD.8.44.KJGXLIVFA*, Δ*phbBAC*) was constructed from strain AZBB721 [41]. This strain also lacks the ammonium transporter AmtB to eliminate the potential for a futile active ammonium pump [42]. The strain was constructed using a parallel approach to that used to insert the *nifA* into an intergenic region of *A. vinelandii* using a combination of the approaches described in [43], [44]. This strain does not produce appreciable levels of PHB or alginate, and yielded similar levels of ammonium to what we previously reported for AZBB664 [43], while accumulating approximately half of the biomass (as measured by optical density of cultures at 600 nm).

## 3 Results and discussion

### 3.1 Initial nutrients and inoculation ratio influence the synthetic co-culture

**Fig. 1:**
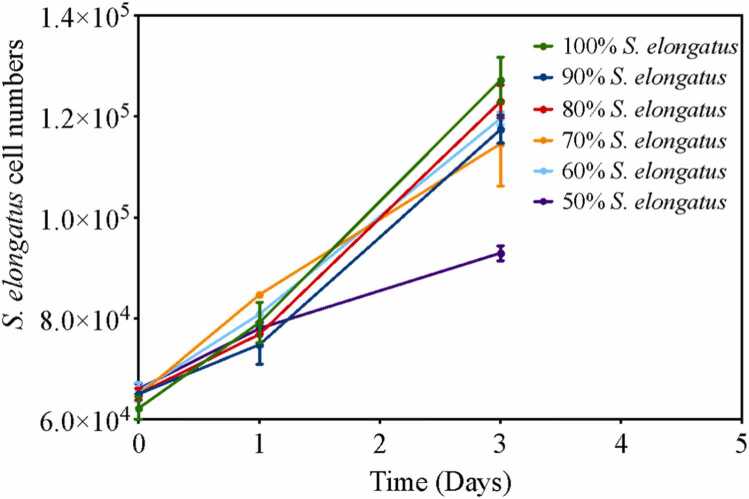
Growth of *S. elongatus* at different inoculation ratios. Ratios are reported as a percentage of *S. elongatus* cell number, e.g., 60 % *S. elongatus* indicates a ratio of 60:40 of *S. elongatus*: *A. vinelandii*. 100 % *S. elongatus* is the control. n = 3. Error bars represent standard deviation.

We began by exploring the optimal starting conditions for our cross-feeding microbial co-culture comprising sucrose-secreting *S. elongatus* [25], [45] and ammonium-secreting *A. vinelandii* [26], where the sucrose produced from *S. elongatus* can be the carbon source for *A. vinelandii* and the ammonium secreted from *A. vinelandii* can be the nitrogen source for *S. elongatus*. To initiate growth in our synthetic microbial co-culture, we first tested co-culture media with different compositions of carbon source, nitrogen source, salt and buffer (Supplementary files, Table S1); we selected nitrate as our initial nitrogen source because it can be readily consumed by *S. elongatus*, but is not taken up and metabolised by *A. vinelandii*, thus not affecting nitrogen fixation. We was discovered that the co-culture required an initial “spike” of carbon and nitrogen sources within the growth media to support growth (Supplementary files, Figure S1). The initial inoculation ratio of each cell type is another key consideration when constructing synthetic co-cultures, as this can greatly influence the growth dynamics of the consortium, as well as its metabolic capacity [45]. Considering that the growth rate of *S. elongatus* is slower than that of *A. vinelandii* (Supplementary files, Table S2 and Figure S2), we examined initial inoculation ratios with higher cell number proportions of *S. elongatus* compared to *A. vinelandii*, namely 50 %, 60 %, 70 %, 80 %, 90 %, and 100 % *S. elongatus*. The 100 % *S. elongatus* condition represented the monoculture control for this experiment. Although a statistically significant difference in growth rates was not observed, the *S. elongatus* initial ratio of 80 % was the most similar rate to *S. elongatus* monoculture over three days (Fig. 1; Supplementary files, Table S3) and was therefore used as the initial *S. elongatus*: *A. vinelandii* inoculation ratio (80:20) for subsequent experiments.

### 3.2 A comparison of co-cultures to monocultures

**Fig. 2:**
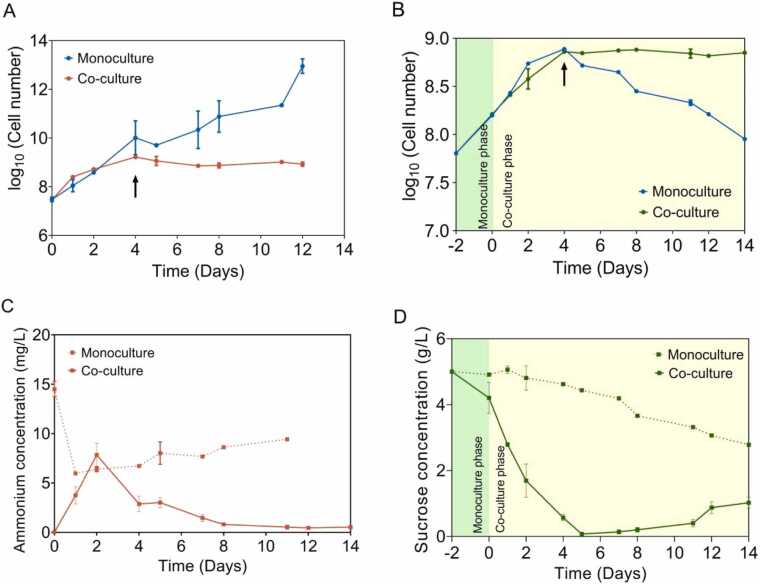
Growth and production of each strain in co-culture and monoculture conditions. (A) Growth curve of *A. vinelandii* in co-culture (red) and monoculture (blue). (B) Growth curve of *S. elongatus* in co-culture (green) and monoculture (blue). (C) Ammonium concentrations in co-culture (solid line) and *A. vinelandii* monoculture (dashed line). (D) Sucrose concentrations in co-culture (solid line) and in *S. elongatus* monoculture (dashed line). IPTG-induced *S. elongatus* was cultured axenically for two days (green shading) before inoculation with *A. vinelandii* on Day 0, establishing the co-culture (yellow shading). The co-cultures were primed with 5 g/L sucrose and 0.4 g/L sodium nitrate. n = 3. Error bars represent standard deviation.

Next, we compared the growth and nutrient dynamics of *S. elongatus* and *A. vinelandii* co-cultures with their respective monocultures. *S. elongatus* and *A. vinelandii* co-cultures were grown in SAC medium with an initial cell ratio of 80:20. Carbon secreted by *S. elongatus* and/or sucrose from the SAC media promoted the growth of the co-cultured heterotroph for the first two days (Fig. 2 A). However, *S. elongatus* cell numbers reduced in the monoculture after initial nitrogen source depletion (Fig. 2 B). The lower concentrations of sucrose and ammonium (Fig. 2 C and D) in co-culture compared to monoculture implies uptake by the corresponding partner strain.

From these growth dynamics, growth of *A. vinelandii* in co-culture slows from day two onwards, although the cell density is maintained for the remaining 10 days. Conversely, *A. vinelandii* in monoculture continues to grow over the same time period. We hypothesise that *A. vinelandii* cells could have become carbon limited in the co-culture, although there might be competition for other micronutrients or other interaction processes slowing growth. The rapid decline in sucrose in the media up to day 5 (Fig. 2 D) in co-culture does imply potential carbon limitation. *S. elongatus* grows more rapidly in monoculture compared to co-culture, although after day 4, cell densities begin to reduce. However, in co-culture, growing cells reach a steady cell density from day 4–14, presumably consuming the ammonia being produced by its partner. It is possible that *S. elongatus* cells in monoculture become nitrogen limited as ammonia concentrations are low. It should be noted that measuring the concentrations of sucrose and ammonia in the media does not reveal production or uptake rates, rather they provide a snapshot of current levels in the media.

To understand these complex dynamics in more detail, the time point of Day 4 (Fig. 2 A and B) was chosen to perform quantitative proteomics. At this time point, *A. vinelandii* in the monoculture is growing steadily, while the cell numbers in the co-culture appear to have stabilized. Although the cell densities of *S. elongatus* are similar in mono and co-cultures on Day 4, the cell numbers decline rapidly in the *S. elongatus* monoculture for the remainder of the experiment.

### 3.3 Overview of the co-culture proteome determined by label-free quantitative analysis

To test the feasibility of using label-free proteomics to analyse the global proteome of *S. elongatus* and *A. vinelandii* in co-culture, we previously carried out a series of preliminary experiments and analyses, which lead to the development of a normalisation method termed LFQRatio [23]. Three biological replicates of both *S. elongatus* and *A. vinelandii* monocultures, as well as the established co-culture, were grown in SAC medium. Samples were harvested on Day 4 and the proteins extracted, processed and analysed for LFQ proteomics. A total of 13552 tryptic peptides from *A. vinelandii* and 15003 peptides from *S. elongatus* were identified across all biological and technical replicates with 1 % FDR, corresponding to 1571 proteins of *A. vinelandii* and 1651 proteins of *S. elongatus* with two or more unique peptides. Proteome coverages of 31.3 % for *A. vinelandii* (5013 proteins) and 57.4 % for *S. elongatus* (2876 proteins) were achieved, which are comparable to other proteomics analyses of the microorganisms [46], [47], [48], [49].

To mitigate biases inherent to co-culture proteomics datasets, we applied additional filtering steps prior to differential expression analysis. In monoculture, protein detection coverage was high for both organisms, with 87.0 % and 89.8 % of identified proteins detected for *A. vinelandii* and *S. elongatus*, respectively (Supplementary files, Figure S3). In contrast, coverage was substantially reduced in co-culture, with only 58.8 % of identified *A. vinelandii* proteins and 67.7 % of identified *S. elongatus* proteins detected (Supplementary files, Figure S3). Reduced protein detection in co-cultures is a significant challenge in metaproteomics and mixed-species proteomics experiments, particularly when large differences in cell numbers or proteome complexity exist between conditions, leading to increased stochasticity in peptide ion selection during MS/MS acquisition [24], [50]. Under these circumstances, missing values are predominantly missing at random, arising from altered sampling depth as opposed to true biological absence. As we cannot effectively determine biologically meaningful absences from stochastic missingness, proteins that were completely absent from either condition were excluded from further analysis. Additionally, proteins were required to be present in at least two out of three biological replicates in one condition to ensure quantitative robustness. Following these filtering steps, 877 and 1107 proteins were quantified for *A. vinelandii* and *S. elongatus*, respectively (Supplementary files, Table S5 and S6).

**Fig. 3:**
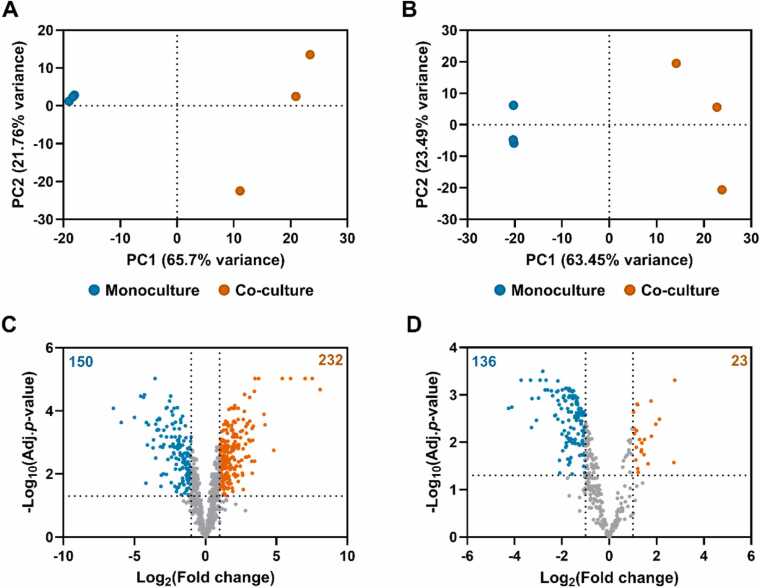
Comparison of *A. vinelandii* and *S. elongatus* proteomes in co-culture and monoculture conditions. (A) and (B): Principal component analyses of LFQ intensity data for (A) *A. vinelandii* and (B) *S. elongatus* for three biological replicates. (C) and (D): Volcano plots showing significantly enriched (orange) and reduced (blue) proteins in co-culture compared to monoculture for (C) *A. vinelandii* and (D) *S. elongatus*. The dotted lines represent |Log_2_FC|= ≥ 1 on the x-axis and adjusted *p*-value cut-off of 0.05 on the y-axis.

Principal component analyses of the proteomic data for both organisms show clear clustering of monocultures separate from co-cultures at Day 4 (Fig. 3A, B). In *A. vinelandii*, of the 877 quantified proteins analysed with Amica [39], 232 proteins were significantly more abundant and 150 proteins significantly less abundant (|Log_2_FC|= ≥ 1, *p*-value < 0.05) in co-culture compared to monoculture (Fig. 3C). Proteomic changes were also evident in *S. elongatus*, in which 23 proteins were more abundant, and 136 proteins less abundant, in co-culture compared to monoculture (Fig. 3D). Overall, these results implicate widespread proteomic changes in response to growth with their cross-feeding partner.

**Fig. 4:**
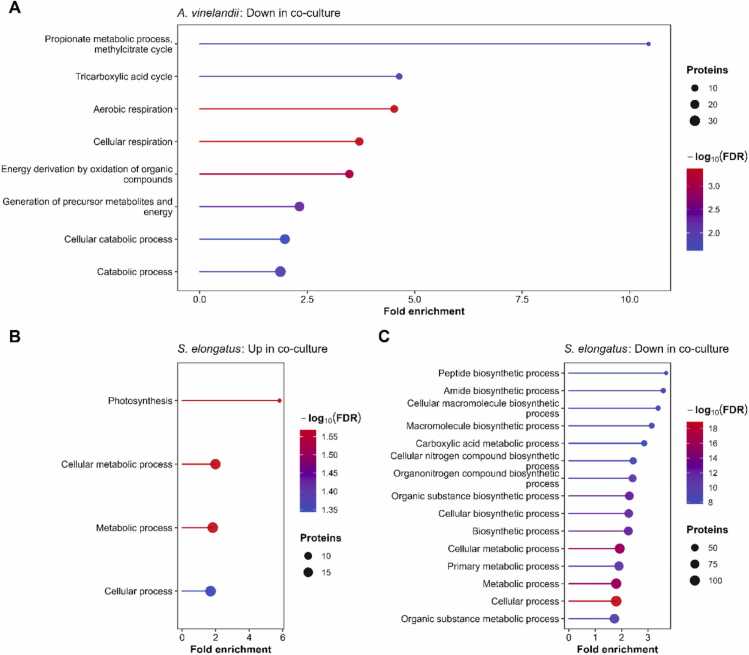
Enriched biological process GO (GO-BP) terms of differentially expressed proteins in co-culture compared to monoculture on Day 4. (A) *A. vinelandii* GO-BP terms significantly less enriched in co-culture compared to monoculture. (B) and (C): *S. elongatus* GO-BP terms significantly (B) more enriched and (C) less enriched in co-culture compared to monoculture. Proteins represents the number of proteins associated with each GO-BP term. Top 15 GO-BP terms ranked by FDR were displayed. FDR = false discovery rate. Significance cut-offs for differential expression: |Log_2_FC|= ≥ 1; adjusted *p*-value < 0.05.

To determine how the metabolisms of *S. elongatus* and *A. vinelandii* differed between co-culture and monocultures, we employed gene ontology (GO) analysis to understand which cellular processes and metabolic pathways were affected by growth in co-culture (Fig. 4). Biological processes less enriched in *A. vinelandii* co-culture compared to monoculture include propionate metabolism, tricarboxylic acid (TCA) cycle, aerobic respiration, and cellular respiration, suggesting diversion of carbon resources from the TCA cycle in the co-culture, and more active respiration and growth in the monoculture, as indicated in the growth data (Fig. 4A; Fig. 2A). Functional analysis and enrichment of *S. elongatus* DEPs revealed that proteins involved in photosynthesis and cellular metabolic processes were enriched in co-culture compared to monoculture, showing active light harvesting and metabolism in co-culture (Fig. 4B). Proteins involved in protein biosynthesis, carboxylic acid metabolism and organonitrogen compound biosynthesis are less enriched in co-culture compared to monoculture, which suggests *S. elongatus* cells are actively producing nitrogen-rich compounds in the monoculture immediately before the culture begins to crash (Fig. 4C, Fig. 2B).

### 3.4 Proteome changes in *A. vinelandii* co-culture compared with monoculture

**Fig. 5:**
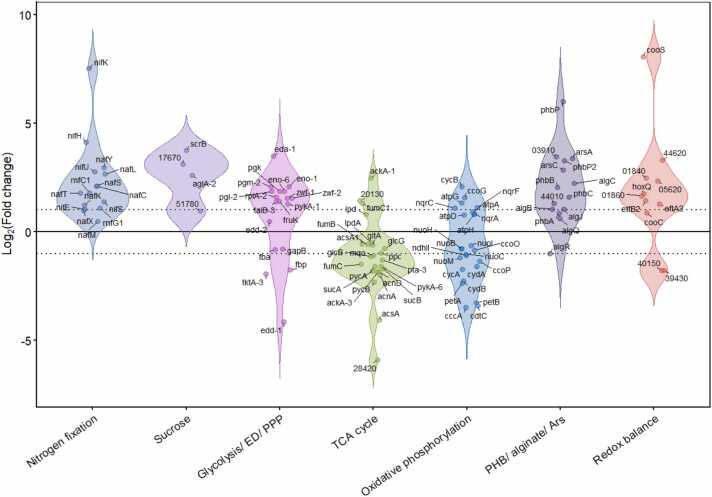
Violin plot showing Log_2_FC fold changes in proteins in key metabolic pathways in *A. vinelandii* co-culture compared to monoculture on Day 4. Number labels represent gene designations without the “Avin_” suffix. ED, Entner–Doudoroff pathway; PPP, pentose phosphate pathway; TCA, tricarboxylic acid cycle; PHB, β-polyhydroxybutyrate; Ars, alkylresourcinols. Dotted lines show |Log_2_FC|= ≥ 1 cut-off. Adjusted p-value < 0.05 for all proteins plotted.

In *A. vinelandii*, various metabolic processes were affected by the presence of its co-culture partner *S. elongatus*, one of the most prominent differences being an increase in abundance of proteins involved in nitrogen fixation in co-culture compared to monoculture (Fig. 5A). Strong relative upregulation was observed in several nitrogenase subunits (nifK, nifH, nifU, nifS, nifE), as well as a nitrogenase-associated respiratory complex (rnfC1, rnfG1), in co-culture compared to monoculture (Fig. 5). The nitrogenase protein is a key protein for nitrogen fixation, which contains two components, component I (Fe-Mo protein) encoded by *nifD* and *nifK* genes and component II (Fe protein) encoded by *nifH* gene [51]. The higher abundance of these proteins indicates an increased requirement of nitrogen during co-culture with *S. elongatus*. As ammonium is a shared resource between *A. vinelandii* and *S. elongatus* in the co-culture, this may explain the observed reduction in extracellular ammonium concentration compared to monoculture (Fig. 1C); here, *S. elongatus* may be acting as an ammonium “sink”, triggering an increase in expression of nitrogen fixation proteins in co-cultured *A. vinelandii*.

Proteins involved in sucrose utilisation (scrB, aglA-2 and Avin_17670) were significantly more abundant in co-culture compared to monoculture (Fig. 5), which implies active uptake and utilisation of sucrose secreted by *S. elongatus* in the co-culture. Likewise, enzymes of the oxidative pentose phosphate pathway (zwf-1, zwf-2, pgl-2) and glycolysis (pgk, eno-1/6, pgm-2, pykA-1) were overall more abundant in co-culture (Fig. 5), providing evidence that sucrose-derived carbon was channelled into central metabolism. However, the Entner–Doudoroff pathway appeared to bifurcate; 2-dehydro-3-deoxy-phosphogluconate aldolase (eda-1) was more abundant in co-culture (log2FC 3.50), while phosphogluconate dehydratase (edd-1) was strongly downregulated (log2FC −4.16), suggesting carbon flux rerouting, potentially reflecting redox or regulatory constraints under co-culture conditions.

As indicated by the GO term enrichment analysis, enzymes associated with the TCA cycle were less abundant in co-culture compared to monoculture (Fig. 4A; Fig. 5), indicating reduced flux through central oxidative metabolism. In parallel, proteins involved in PHB biosynthesis (phbA, phbB, phbC) and PHB granule-associated phasins (phbP, Avin_03930) were more abundant. In *A. vinelandii*, PHB synthesis is initiated by phbA, which condenses two molecules of acetyl-CoA to form acetoacetyl-CoA, thereby directly competing with the TCA cycle for carbon flux [52]. The reduced TCA cycle and increased PHB-related proteins suggest that acetyl-CoA derived from sucrose metabolism was diverted towards carbon storage, rather than complete oxidation, in co-culture. However, PHB quantification by GC-MS of co-culture biomass harvested on Day 4 revealed extremely low PHB levels (0.44 % DCW; Supplementary files, Figure S4). This apparent discrepancy is likely explained by the simultaneous enrichment of two PHB depolymerases (Avin_03910, Avin_44010), suggesting active PHB turnover rather than net storage [53], [54]. PHB biosynthesis and depolymerization are tightly regulated in *A. vinelandii*, and the concurrent induction of both processes may indicate redox or oxygen stress that is rapidly alleviated within sub-populations of cells within the co-culture [55]. Consistent with this interpretation, the stationary-phase sigma factor rpoS (log2FC 3.00), which positively regulates PHB synthesis and encystment in *A. vinelandii*, was significantly more abundant in co-culture, indicating a regulatory shift towards a stress-adapted physiological state [56], [57].

Proteins involved in energy production and conversion were less abundant in *A. vinelandii* co-culture, which corresponds with its slower growth rate compared to monoculture at Day 4 (Fig. 2A). Key proteins in oxidative phosphorylation, including subunits of NADH-dehydrogenase I (nuoC, nuoM), NADH-dehydrogenase II (ndhII), cytochrome electron carriers (petA, petB, cycA), and terminal oxidases (cydA, cydB, ccoP) [58], [59], [60] were less abundant in co-culture, suggesting that less biosynthetic processes were powered by the oxidative phosphorylation pathway (Fig. 5). At the same time, several less well-characterised respiratory chain components were significantly enriched in co-culture, including subunits of an Na⁺-translocating NADH:quinone reductase (nqrC, nqrF), an electron transfer flavoprotein (etfA2, etfB2), NAD(P) transhydrogenase (Avin_01840, Avin_01860), and an FAD-dependent oxidoreductase (Avin_44620), which suggests *A. vinelandii* undergoes bioenergetic rewiring in response to redox changes elicited by growth alongside *S. elongates* [61].

Other proteins involved in redox balancing and oxidative stress were also strongly induced in co-culture, including alkyl hydroperoxide reductase (Avin_26320), superoxide dismutase (sodB), and glutathione-associated enzymes which, along with the widespread downregulation of terminal oxidases, suggests that *A. vinelandii* undergoes increased oxidative or redox stress in co-culture, potentially due to elevated oxygen levels resulting from photosynthetic activity of *S. elongatus* (Fig. 5). Notably, the highest relative protein abundance change in *A. vinelandii* during co-culture was for a carbon monoxide (CO) dehydrogenase subunit (*cooS*; log_2_FC 8.08), which putatively converts CO to CO_2_ [62], [63]. CO is a potent inhibitor of nitrogen fixation [64], so cooS may be upregulated as a protective mechanism to protect the overexpressed nitrogenase during redox-imbalanced conditions triggered by *S. elongatus*.

Two-component signal transduction systems enable bacteria to sense, respond, and adapt to changes in the environment or intracellular state [65]. We observed multiple two-component systems that changed in the synthetic co-culture. Among them, phosphate sensing and transport proteins phoR (log_2_FC 1.42) and phoU (log_2_FC 1.67) exhibited higher relative abundance in *A. vinelandii*, suggesting phosphate acquisition is prioritised in the co-culture medium [66]. TonB-dependent ferrisiderophore receptor protein (pfeA, log_2_FC 1.75), involved in the transport of siderophores into the periplasm, was also more abundant in *A. vinelandii* co-culture, indicating iron limitation and competition between the two organisms when grown together [67].

### 3.5 Proteome changes in *S. elongatus* co-culture compared with monoculture

**Fig. 6:**
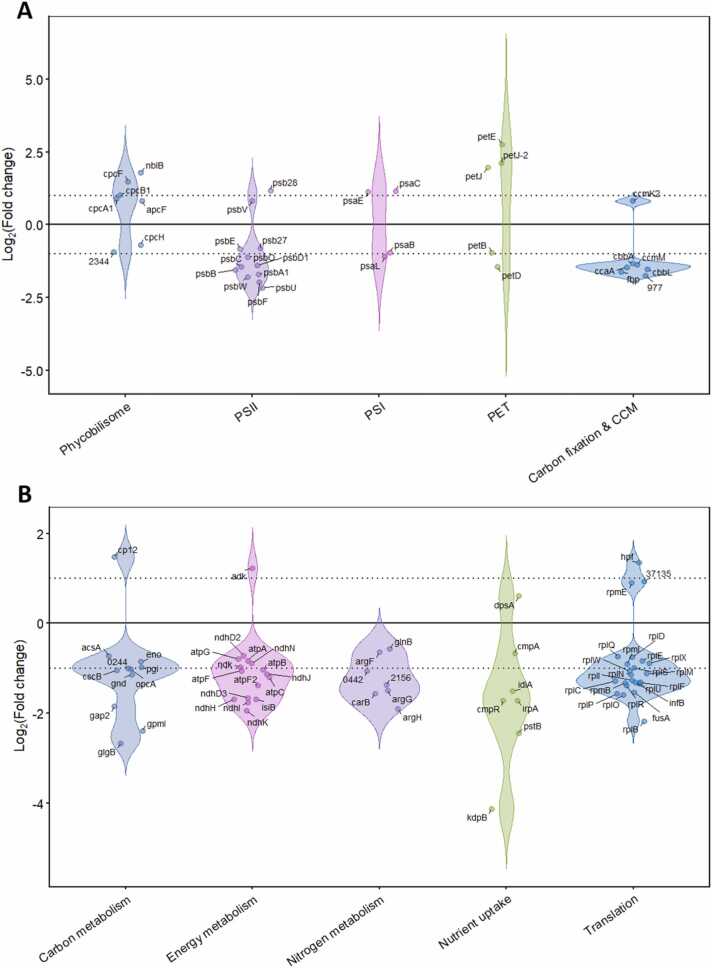
Metabolic changes in *S. elongatus* co-culture compared to monoculture on Day 4. Violin plots showing the Log_2_FC of proteins in (A) photosynthesis and (B) key metabolic pathways and processes in *S. elongatus*. Number labels represent gene designations following “Synpcc7942_” suffix. PSII, photosystem II; PSI, photosystem I; PET, photosynthetic electron transport; CCM, carbon concentrating mechanism. Dotted lines show |Log_2_FC|= ≥ 1 cut-off. Adjusted *p*-value < 0.05 for all proteins plotted.

To better elucidate the metabolic pathways underpinning the sustained *S. elongatus* growth in co-culture compared to monoculture, we compared the proteomes of *S. elongatus* grown in co-culture and monoculture at Day 4. The production and secretion of sucrose from *S. elongatus* is highly dependent on efficient photosynthesis and carbon fixation, processes for which we observed several distinctions between monoculture and co-culture conditions (Fig. 6A). Several phycobilisome antenna proteins (cpcB1, cpcF, nblB) were more abundant in co-culture, suggesting that *S. elongatus* was effectively harvesting light; however, multiple components of photosystem II (PSII) were less abundant in co-culture compared to monoculture (Fig. 6A). Regulation of PSI appeared mixed, with some subunits upregulated in co-culture (psaC, psaE), which suggests re-routing of electron flow towards cyclic electron transfer. Supporting this, mobile electron carriers including plastocyanin (petE) and two cytochrome c6 proteins (petJ and petJ-2), which shuttle electrons to the PSI complex, were also more abundant [68]. Cyclic electron flow around PSI maintains ATP generation without generating NADPH or evolving O_2_, indicating that *S. elongatus* is modulating its cellular reducing power in response to growth with *A. vinelandii* [69](Fig. 6A). In addition, several CO_2_ fixation and carbon-concentrating mechanism proteins were less abundant in *S. elongatus* co-culture (Fig. 6A). This could be due to reduced emphasis on rapid growth, consistent with the growth data (Fig. 2B), but could also indicate a response to increased ammonia availability, balancing the cellular C/N ratio, redox balancing to limit demand for reducing equivalents, or reduced CO_2_ limitation in co-culture due to its proximity to respiring *A. vinelandii* generating CO_2_.

Consistent with the down-tuning of carbon fixation, enzymes associated with central carbon metabolism were overall less abundant in *S. elongatus* co-culture compared to monoculture (Fig. 6B). Proteins involved in glycogen metabolism (glgB, Synpcc7942_0244), glycolysis, and the pentose phosphate pathway (gap2, gpmI, gnd, opcA) were all less abundant in co-culture (Fig. 6B). The CP12 protein (Synpcc7942_0361), a negative regulator of the Calvin-Benson cycle and the oxidative pentose phosphate pathway in cyanobacteria [70], was also enriched in co-culture, further supporting alterations in redox balance. Notably, IPTG-induced overexpression of recombinant sucrose permease (cscB) was reduced in co-culture compared to monoculture, suggesting that less sucrose is transported out of *S. elongatus* cells; this likely a consequence of broader downregulation of translation in co-culture (Fig. 6B). Moreover, ATP synthase subunits (atpB, atpC, atpF, atpF2) and NDH-1 complex subunits (ndhD3, ndhH, ndhI, ndhJ, ndhK) were less abundant in co-culture compared to monoculture. Collectively, these changes indicate a de-prioritisation of growth and maintenance of a low-flux steady state in co-culture, as observed in the growth data (Fig. 2B; Fig. 6B). This likely contributes to the long-term stability of *S. elongatus* when grown with *A. vinelandii*.

Given the limited amount of nitrate available to *S. elongatus* in the initial SAC medium (0.4 g/L), the observed decline in *S. elongatus* growth after Day 4 in monoculture can likely be attributed to nitrogen deficiency (Fig. 2B). This assumption is supported by the increased abundance of nitrogen uptake and assimilation proteins in the monoculture, such as an ammonium transporter (Synpcc7942_0442), glutamine synthetase (Synpcc7942_2156) and carbamoyl-phosphate synthase large subunit (carB) (Fig. 6B). Nitrogen regulatory protein P-II (glnB) was also less abundant in co-culture, although not significantly so (Fig. 6B). This suggests that co-cultured *S. elongatus* cells were nitrogen replete relative to the monoculture.

In addition to the ammonium transporter, several other uptake transporters were relatively reduced in co-culture, implicating increased demand for phosphate (ptsB), iron (idiA, irpA), and inorganic carbon (cmpR) in the *S. elongatus* monoculture (Fig. 6B), which suggests that *S. elongatus* cells in monoculture are beginning to scavenge nutrients as they reach the end of exponential growth (Fig. 2B).

### 3.6 Co-culture analysis over time

**Fig. 7:**
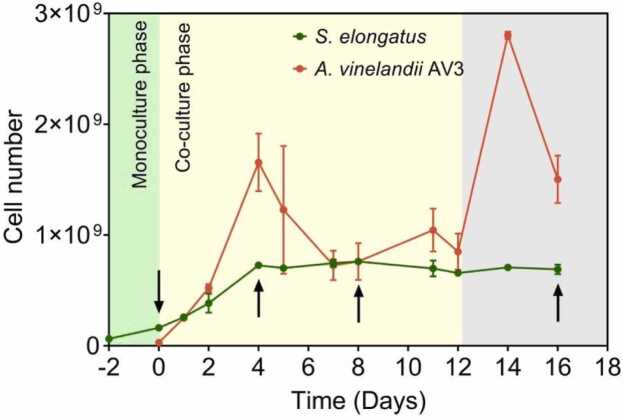
Growth curves of *S. elongatus* (green line) and *A. vinelandii* AV3 (red line) in co-culture. *A. vinelandii* AV3 was inoculated into *S. elongatus* culture two days after IPTG induction. *S. elongatus* cell counting was achieved using flow cytometric analysis. *A. vinelandii* cell counting was achieved by CFUs assay, subsequently converted to cell numbers via a CFUs-cell number standard curve. *A. vinelandii* cell shape changes after 12 days, which affects the accuracy of *A. vinelandii* cell counts (grey shading). The arrows indicate when proteomic samples were taken.

Due to the complex interplay of these microbes in co-culture, and the caveats of omics experiments when only one time point is considered, a co-culture proteomics experiment was undertaken over a time course (Fig. 7).

*A. vinelandii* grew steadily over the first four days of co-culture (Fig. 7), consuming sucrose from the media as a carbon source. After this period, *A. vinelandii* growth slowed, but a second increase was seen after Day 8, likely due to higher sucrose levels being provided by its microbial partner. As an ammonium producer, *A. vinelandii* caused extracellular ammonium concentrations to rise during rapid growth from 0 mg/L to a maximum of 7.84 mg/L on Day 2, then gradually decline (Fig. 2C). Meanwhile, *S. elongatus* cells grew rapidly for four days, which was accompanied by a decrease in ammonium in the media, suggesting that *S. elongatus* was using ammonium as a nitrogen source after starting with nitrate. From Day 4, *S. elongatus* cell numbers stabilized until Day 16. Based on these growth and nutrient patterns, biomass samples were collected on Days 0, 4, 8 and 16 for LC-MS/MS proteomics analysis. Due to the changing cell shape of *A. vinelandii* after 14 days (Supplementary file, Figure S5), the cell number counting method became unreliable for this strain. Therefore, only the first three time points are discussed in detail for *A. vinelandii*.

The first two time points were discussed in our previous study [23], where we focussed on carbon and nitrogen macronutrient exchange. Briefly, MaxQuant identified a total of 1556 proteins in *S. elongatus* and 1158 in *A. vinelandii*. In that analysis, we applied a stringent filtering approach in which all missing values were filtered out prior to differential expression analysis [23]. However, we reduced the stringency for the current analysis by filtering out only proteins that were completely absent from one of the two conditions being compared, or that were quantified in less than two biological replicates in both conditions compared. This approach maximised the number of quantifiable proteins, while still reducing false negatives arising from limited proteome coverage in mixed bacterial samples. Table 1 shows the numbers of quantified, differentially regulated, and more or less abundant proteins for each comparison. Tables showing DEPs for each comparison can be found in Supplementary files, Table S7–S12.

**Table 1:** Comparisons and quantifications of analysed proteins in *A. vinelandii*-*S. elongatus* co-culture over time. D0, D4, D8 and D16 represent Day 0, Day 4, Day 8, and Day 16, respectively. DEPs, differentially expressed proteins. Significance cut-offs: |log2FC= ≥ 1; adjusted p-value < 0.05.

Organism	Comparison	Quantified proteins	Significant DEPs	More abundant in A	More abundant in B
*A. vinelandii*	D4^A^ vs D0^B^	759	99	23	76
	D8^A^ vs D4^B^	755	192	101	91
	D16^A^ vs D8^B^	333	128	59	69
*S. elongatus*	D4^A^ vs D0^B^	1104	456	102	354
	D8^A^ vs D4^B^	1033	118	44	74
	D16^A^ vs D8^B^	1063	28	7	21

From Day 0 to Day 4, five *S. elongatus* photosynthesis proteins increased in abundance, including the plastocyanin (petE, log_2_FC 2.17), photosystem I iron–sulfur center (psaC, log_2_FC 1.53), photosystem I reaction center subunit IV (psaE, log_2_FC 1.46), cytochrome c550 (psbV, log_2_FC 1.42), and photosystem II reaction center Psb28 protein (psb28, log_2_FC 1.07). Additionally, glyceraldehyde-3-phosphate dehydrogenase (log_2_FC 1.26), involved in carbon fixation, was more abundant, suggesting increased carbon assimilation and growth. In the same period, *S. elongatus* L-glutamine synthetase, which converts L-glutamate and ammonia to L-glutamine, rose in abundance (log_2_FC 1.24). Meanwhile, *A. vinelandii* increased its expression of sucrose-6-phosphate hydrolase (*scrB*, log_2_FC 2.64), indicating higher sucrose uptake, consistent with reduced sucrose levels in the media as cell numbers rose. This was accompanied by an increase in PHB biosynthetic enzymes phbA (log_2_FC 1.40) and phbB (log_2_FC 1.09) in *A. vinelandii* from Day 0 to Day 4.

Between Days 4 and 8, there were less pronounced changes in *S. elongatus*, which was unsurprising as cell counts were very similar implying no growth. *S. elongatus* proteins like sucrose phosphate synthase (*sps*, log_2_FC 1.17) increased, showing higher carbon assimilation and sucrose production. L-glutamine synthetase (*Synpcc7942_2296*, log_2_FC 1.18) also increased, showing integration of *A. vinelandii*-secreted ammonium into metabolism.

However, the comparison of Day 8 to Day 4 revealed more intriguing changes in the *A. vinelandii* proteome, indicating more stress as it secreted polysaccharides. A mannuronan C-5 epimerase had the largest increase in abundance (*algE1*, log_2_FC 5.42), which remodels alginate during secretion. The changing of colony morphology may also be explained by the increase in the encystment and alginate biosynthesis response regulator AlgR (*algR*, log_2_FC 2.57). Alginate production has been implicated in protecting *A. vinelandii* and its nitrogenase components from oxygen stress, suggesting the stable photosynthesising *S. elongatus* population is triggering oxygen stress in *A. vinelandii* in co-culture [71]. As well as the production of alginate, there was also evidence for increased PHB production during this period, with a log_2_FC 2.49 increase in the poly (3-hydroxybutryate) synthase subunit. However, the log_2_FC 1.79 increase in a poly (3-hydroxybutryate) depolymerase protein implies a more complex overview of how carbon storage is being controlled in co-culture conditions. It is clearer that *A. vinelandii* is experiencing increasing stress during this time period by synthesising stress proteins such as heat shock protein Hsp20 (log_2_FC 1.90), Universal stress protein (log_2_FC 2.75) as well as other chaperonins (Supplementary files, Table S8).

### 3.7 Alginate/PHB *A. vinelandii* mutant growth comparison

**Fig. 8:**
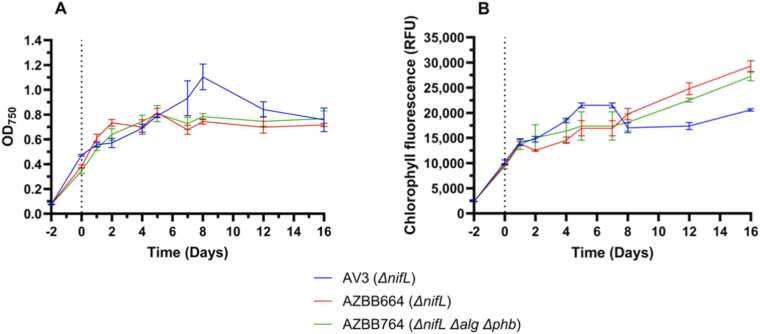
Growth comparison of three *A. vinelandii* strains cultivated in co-culture with *S. elongatus.* (A) Co-culture growth tracked by OD750 measurements of co-cultures grown with the *A. vinelandii* strain (AV3) used in our co-culture proteomics experiments (blue), a more recently generated ammonia secretion strain AZBB664 (Δ*nifLA*, Δ*amtB*; red), as well as AZBB764 (Δ*nifLA*, Δ*amtB*, Δ*algD.8.44.KJGXLIVFA*, Δ*phbBAC*), a version where alginate and PHB synthesis have been deleted (green). (B) The equivalent growth of *S. elongatus* with the above-mentioned *A. vinelandii* strains, tracked by measuring chlorophyll fluorescence.

The co-culture proteomics profile implied carbon stress responses in *A. vinelandii* cells, leading to a complex remodelling of central carbon metabolism, and involving secretion of polysaccharides. A small amount of PHB accumulation was observed at Day 4, which also implies carbon limitation was a factor at some point of the cultivation (Supplementary files, Figure S4). The impact of accumulating or attempting to accumulate biopolymers, or secrete polysaccharides on co-culture growth dynamics is unknown, and therefore a PHB/alginate mutant *A. vinelandii* strain was generated and cultivated in co-culture with *S. elongatus.* The mutant strain was observed to be more translucent than the parent type, implying an altered physiology. However, our growth comparison experiments showed no significant differences when comparing the co-cultures (Fig. 8).

## 4 Conclusions

In this study, a growth analysis of a synthetic microbial co-culture composed of two engineered strains, a carbon-fixing cyanobacterium *S. elongatus* PCC 7942 cscB/SPS and a nitrogen-fixing bacterium *A. vinelandii* AV3, was undertaken in mixed flasks. We confirmed that the initial cell ratio can influence the growth of co-culture, and thus interaction dynamics in the system. Co-cultures could be maintained for up to 14 days, during which *S. elongatus* is able to utilise ammonium from *A. vinelandii* to maintain a steady cell concentration. This contrasts with monocultures of *S. elongatus,* where nitrogen depletion leads to a reduction in cell density from Day 4 onwards. Although *A. vinelandii* can also maintain a steady cell concentration for the 14 days, its change in morphology as well as the ability to continue growth in monoculture beyond 4 days, points to potential stress conditions in co-culture.

By applying a quantitative proteomics pipeline for comparing co-culture to monocultures and a time series of co-culture cultivation, carbon and nitrogen nutrient exchanges supported cross-feeding between the two strains. Complex re-modelling of energy requirements (photosynthesis and central carbon metabolism) hypothesized from growth data were evidenced, with physiological indicators of stress in *A. vinelandii* cells over time. Although the role of biopolymer and alginate synthesis was predicted to affect the co-culture growth instability, changes were not seen in a PHB/alginate mutant cultivated in co-culture. Cultivation strategies to potentially reduce oxygen levels may also assist, through cultivation in continuous systems, using light dark cycles, as well as growing the strains in novel photobioreactors that have membrane separated sections for each engineered strain. This could limit undesired stress responses hypothetically triggered by cell-cell contact that may act on two-component signal transduction systems. Therefore, to increase stability of the co-culture over time by reducing *A. vinelandii* stress, targeted process optimisations are recommended. In all, these strategies could help establish a more robust microbial foundation, paving the way for stable and scalable consortia for bio-production.

## CRediT authorship contribution statement

**Xiaoxia Nina Lin:** Funding acquisition, Conceptualization. **Brett Barney:** Resources, Methodology. **Yanmeng Liu:** Writing – review & editing. **Caroline Evans:** Writing – review & editing, Supervision, Software, Methodology, Data curation. **Josie McQuillan:** Writing – review & editing. **Mengxun Shi:** Writing – review & editing, Writing – original draft, Visualization, Validation, Methodology, Investigation, Formal analysis, Data curation, Conceptualization. **Jagroop Pandhal:** Writing – review & editing, Writing – original draft, Supervision, Software, Resources, Methodology, Funding acquisition, Formal analysis, Conceptualization.

## Declaration of Competing Interest

The authors declare the following financial interests/personal relationships which may be considered as potential competing interests: Jagroop Pandhal reports financial support was provided by Engineering and Physical Sciences Research Council. Jagroop Pandhal reports financial support was provided by US Department of Energy. Mengxun Shi reports financial support was provided by Chinese scholarship council. If there are other authors, they declare that they have no known competing financial interests or personal relationships that could have appeared to influence the work reported in this paper.

## Data Availability

Data will be made available on request.
