# Supplementary material for: Characterising complex metabolic responses in an engineered, cross-feeding microbial co-culture using quantitative proteomics
Source: N Biotechnol. 2026 Jul 25;93:9–20. doi: 10.1016/j.nbt.2026.02.001 (PMC13179865; doi:10.1016/j.nbt.2026.02.001)
Supplement: Supplementary file 1 — Supplementary material [file mmc1.docx]

**Supplementary Information**

**Characterising complex metabolic responses in an engineered, cross-feeding microbial co-culture using quantitative proteomics**

Mengxun Shi^1^, Josie McQuillan^1^, Caroline Evans^1^, Yanmeng Liu^2^, Xiaoxia Nina Lin^2^, Brett Barney^3^, Jagroop Pandhal^1,*^

^1^ School of Chemical, Materials and Biological Engineering, The University of Sheffield, Sheffield, S1 3JD, UK

^2^ Department of Chemical Engineering, University of Michigan, Ann Arbor, MI 48109, USA

^3^ Department of Bioproducts and Biosystems Engineering, University of Minnesota, St. Paul, Minnesota, USA

**Contents**

**Table S1 Co-culture media compositions adjusted from standard Burk’s medium and BG-11 medium. Components are shown in grams per liter**

**Table S2. The growth rate of *S. elongatus* and *A. vinelandii* monocultures in the exponential phase**

**Table S3. The growth rate of *S. elongatus* at different starting ratios in co-culture**

**Table S4 Parameter settings for MaxQuant**

**Figure S1 Growth curve of co-culture under different media compositions. (A) SAC1 medium, (B) SAC2 medium, (C) SAC3 medium, (D) SAC4 medium, (E) SAC5 medium, (F) SAC6 medium, (G) SAC7 medium. Orange lines represent the growth curves of *A. vinelandii* measured at OD600. Green lines represent growth curves of *S. elongatus* measured at OD750. Each experiment was repeated in three biological replicates. Error bars represent standard deviation.**

**Figure S2. Growth curve of *S. elongatus* and *A. vinelandii* monocultures. (A) Growth kinetics of *S. elongatus* monoculture cultured under standard BG11 medium. Growth was measured by optical density at OD750. (B) Growth kinetics of *A. vinelandii* monoculture cultured under standard Burk’s medium. Growth was measured by optical density at OD600.**

**Figure S3: Quality control plots for co-culture vs monoculture proteomics.**

**Figure S4 Gas chromatography (GC) analysis of polymer synthesised in co-culture.**

**Figure S5 Changing cell shape of *A. vinelandii* after 14 days.**

## Table S1 Co-culture media compositions adjusted from standard Burk’s medium and BG-11 medium. Components are shown in grams per liter

| Media composition (g/L) | | SAC1 | SAC2 | SAC3 | SAC4 | SAC5 | SAC6 | SAC7 |
| --- | --- | --- | --- | --- | --- | --- | --- | --- |
| Carbon source | Sucrose |  |  |  | 5 | 5 | 5 | 5 |
| Nitrogen source | NaNO_3_ |  |  | 0.06 | 0.06 | 0.06 | 0.06 | 0.4 |
| Burk’s phosphate buffer | KH_2_PO_4_ | 0.2 | 0.2 | 0.2 | 0.2 | 0.2 | 0.2 | 0.2 |
|  | K_2_HPO_4_ | 0.8 | 0.8 | 0.8 | 0.8 | 0.8 | 0.8 | 0.8 |
| Burk’s salts | CaCl_2_•2H_2_O | 0.09 | 0.09 | 0.09 | 0.09 | 0.09 | 0.09 | 0.09 |
|  | MgSO_4_•7H_2_O | 0.2 | 0.2 | 0.2 | 0.2 | 0.2 | 0.2 | 0.2 |
|  | Na_2_MoO_4_•2H_2_O | 0.26 | 0.26 | 0.26 | 0.26 | 0.26 | 0.26 | 0.26 |
|  | FeSO_4_•7H_2_O | 0.005 | 0.005 | 0.005 | 0.005 | 0.005 | 0.005 | 0.015 |
| BG-11 stock solutions | K_2_HPO_4_•3H_2_O |  |  |  | 0.04 | 0.04 | 0.2 | 0.2 |
|  | MgSO_4_•7H_2_O | 0.075 | 0.075 | 0.075 | 0.075 | 0.075 | 0.375 | 0.375 |
|  | CaCl_2_•2H_2_O | 0.036 | 0.036 | 0.036 | 0.036 | 0.036 | 0.036 | 0.036 |
|  | Citric acid | 0.006 | 0.006 | 0.006 | 0.006 | 0.006 | 0.006 | 0.006 |
|  | EDTANa_2_•2H_2_O | 0.011 | 0.011 | 0.011 | 0.011 | 0.011 | 0.011 | 0.011 |
|  | Na_2_CO_3_ | 0.02 | 0.02 | 0.02 | 0.02 | 0.02 | 0.02 | 0.02 |
|  | Ammonium ferric citrate green |  |  |  |  | 0.006 | 0.006 | 0.006 |
| BG-11 trace element |  | 1 mL | 1 mL | 1 mL | 1 mL | 1 mL | 1 mL | 1 mL |
| pH buffer | NaHCO_3_ |  | 2 | 2 |  |  |  |  |
|  | HEPES |  |  |  |  | 2.383 | 2.383 | 2.383 |

Composition of BG-11 trace element solution.

| Composition | Concentration (g/L) |
| --- | --- |
| H_3_BO_3_ | 2.86 |
| MnCl_2_•4H_2_O | 1.81 |
| ZnSO_4_•7H_2_O | 0.22 |
| Na_2_MoO_4_•2H_2_O | 0.39 |
| CuSO_4_•5H_2_O | 0.08 |
| Co(NO_3_)_2_•6H_2_O | 0.05 |

## Table S2. The growth rate of *S. elongatus* cscB/SPS and *A. vinelandii* AV3 monocultures in the exponential phase

| Strains | *S. elongatus* cscB/SPS | *A. vinelandii* AV3 |
| --- | --- | --- |
| Specific growth rate (day^-1^) | 0.214 ± 0.079 | 1.803 ± 0.026 |
| Doubling time (days) | 3.655 ± 1.707 | 0.384 ± 0.006 |

Specific growth rate was calculated using equation $\mu=\frac{{ln}_{OD2}-{ln}_{OD1}}{t_{2}-t_{1}};$doubling time was calculated using equation $t_{d}=\frac{ln2}{\mu}$*. S. elongatus* cscB/SPS samples were taken at day 5 and day 7 time points. *A. vinelandii* AV3 samples were taken at day 2 and day 3 time points

## Table S3. The growth rate of *S. elongatus* cscB/SPS at different starting ratios in co-culture

| Starting ratio | 100% *S. elongatus* | 90% *S. elongatus* | 80% *S. elongatus* | 70% *S. elongatus* | 60% *S. elongatus* | 50% *S. elongatus* |
| --- | --- | --- | --- | --- | --- | --- |
| Specific growth rate (day^-1^) | 0.238 | 0.197 | 0.212 | 0.190 | 0.201 | 0.114 |

Specific growth rate was calculated using equation $\mu=\frac{{ln}_{cell number2}-{ln}_{cell number1}}{t_{2}-t_{1}}$

## Table S4 Parameter settings for MaxQuant

| Grouping | Parameters | Settings |
| --- | --- | --- |
| Label-free quantification | Variable modification | Oxidation (M), Acetyl (Protein N-term) |
|  | Fixed modification | Carbamidomethyl (C) |
|  | LFQ minimum ratio count | 2 |
|  | FastLFQ | on |
| Digestion | Digestion Enzyme | Trypsin/P |
|  | Maximum missed cleavages | 2 |
| Instrument | Instrument type | Orbitrap |
|  | First search peptide tolerance | 20 ppm |
| Sequences | Minimum peptide length for unspecific search | 8 |
|  | Maximum peptide length for unspecific search | 25 |
| Quantification | Peptides for quantification | Unique and razor |
| MS/MS analyser | FTMS MS/MS match tolerance | 20 ppm |
|  | ITMS MS/MS match tolerance | 0.5 Da |
| Identification | PSM FDR | 0.01 |
|  | Protein FDR | 0.01 |
|  | Minimum peptides | 1 |
|  | Minimum razor and unique peptides | 1 |
|  | minimum score for modified peptides | 40 |

## Figure S1 Growth curve of co-culture under different media compositions. (A) SAC1 medium, (B) SAC2 medium, (C) SAC3 medium, (D) SAC4 medium, (E) SAC5 medium, (F) SAC6 medium, (G) SAC7 medium. Orange lines represent the growth curves of *A. vinelandii* measured at OD_600_. Green lines represent growth curves of *S. elongatus* measured at OD_750_. Each experiment was repeated in three biological replicates. Error bars represent standard deviation.

## Figure S2. Growth curve of *S. elongatus* and *A. vinelandii* monocultures. (A) Growth kinetics of *S. elongatus* monoculture cultured under standard BG11 medium. Growth was measured by optical density at OD_750_. (B) Growth kinetics of *A. vinelandii* monoculture cultured under standard Burk’s medium. Growth was measured by optical density at OD_600_.

## Figure S3: Quality control plots for co-culture vs monoculture proteomics. A) & B): Number of MaxQuant-identified proteins for each replicate based on LFQ intensities prior to filtering for A) *A. vinelandii* and B) *S. elongatus*. C) & D): The percentage of missing values for each replicate based on LFQ intensities prior to filtering for C) *A. vinelandii* and D) *S. elongatus*. E) & F): Venn diagrams showing overlapping and unique proteins in co- and monoculture prior to filtering for E) *A. vinelandii* and F) *S. elongatus*. Proteins were counted as present if > 0 LFQ intensity values were recorded per condition.


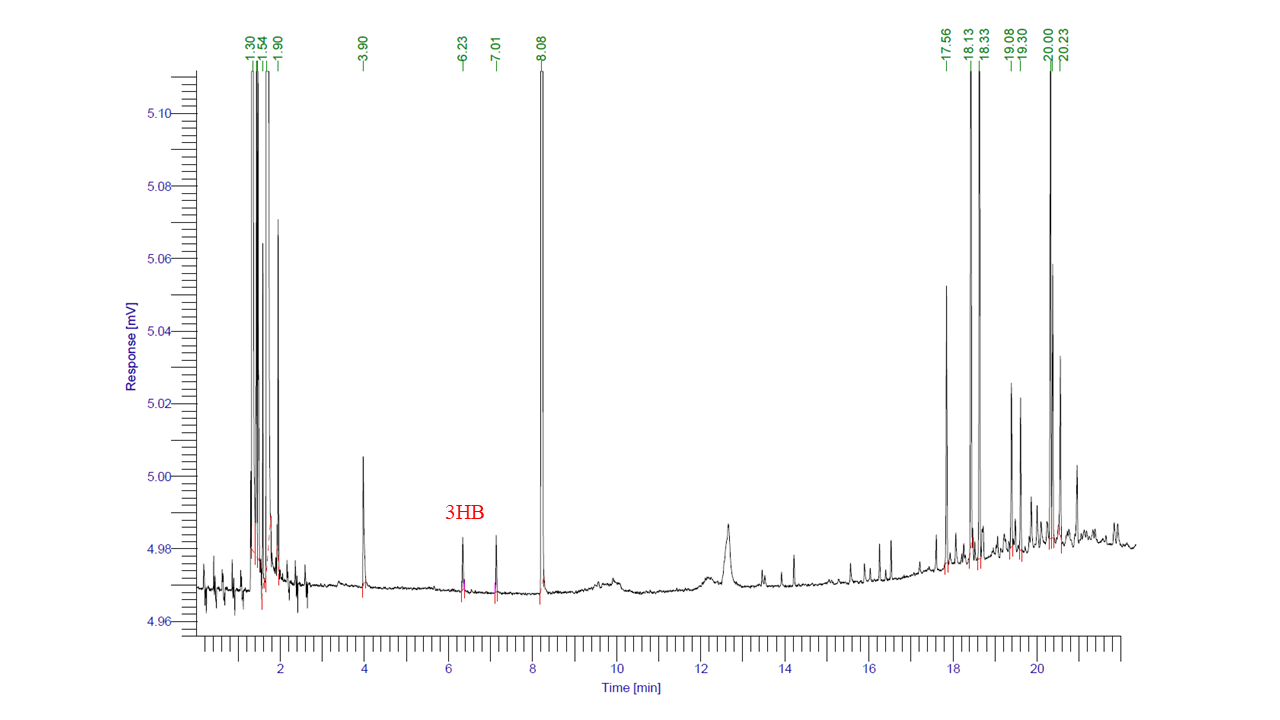


## Figure S4 Gas chromatography (GC) analysis of polymer synthesised in co-culture.

For PHB extraction, approximately 10-20 mg freeze-dried co-culture biomass mixed with 2 mL chloroform and methanolic sulphuric acid. For depolymerization and methanolysis of PHB, the mixture was heated at 100˚C for 4 h with occasional shaking followed by subsequently cooling at room temperature and adding 2 mL of distilled water. The organic layer at the bottom part was collected by a glass Pasteur pipette and subjected to spectral analysis.

Samples were analysed by GC (Perkin Elmer AutoSystem XL Autosampler) equipped with Zebron ZB-5plus Capillary GC column. Samples (0.5 μL) were injected with a sampling rate of 6.25 pts/s. Hydrogen was used as the carrier gas. The oven temperature of the column was programmed from 60 °C for 3 min, then ramped at a rate of 10 °C /min to 230 °C and held at this temperature for 2 min. Methyl benzoate was used as the internal standard.


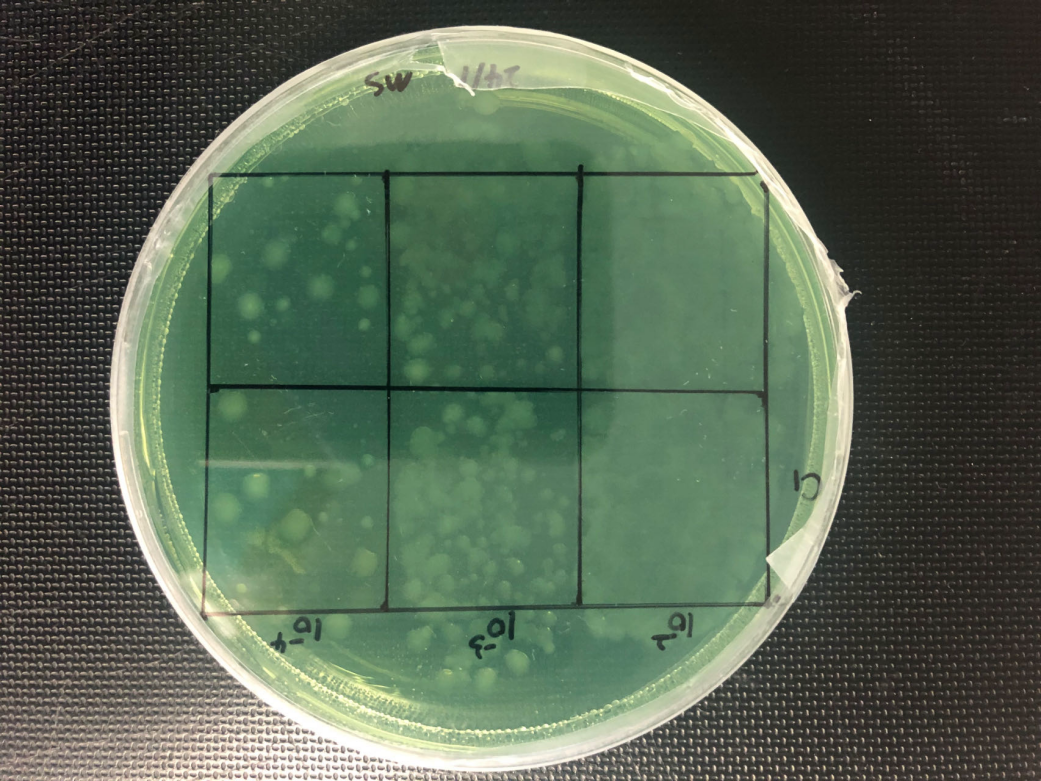


## Figure S5 Changing morphology of *A. vinelandii* after 14 days. 10 μL diluted (10^-2^, 10^-3^, and 10^-^4) *A. vinelandii* samples were plated on the Burk’s agar plate.
